# The Magdalen Hospital, Streatham

**Published:** 1893-11-11

**Authors:** 


### EDITOR'S LETTER-BOX.

[Our correspondents are reminded that prolixity is a great bar to publication, and that brevity of style and conciseness of statement greatly facilitate early insertion.]

### THE MAGDALEN HOSPITAL, STREATHAM.

The Reverend W. Watkins writes: As the warden of the Magdalen Hospital, Streatham, may I ask you to kindly insert in your next issue a few remarks on the paragraph that appeared in the "Notes and News" column of last week? Firstly, this institution is incorporated under an Act of Parliament (9 George III., cap. 31), in which it is provided that there shall be four General Courts of Governors held every year, the one held in the month of April to be the "Annual General Court," and that "notice of all General Courts must be given in the public newspapers at least five clear days before holding the same." It will be seen by this that the managers have no option in the matter, and therefore cannot be blamed or otherwise in regard to "waste of funds" in advertising. Secondly, the Courts are not public in the ordinary sense of the word, as no one has any voice in the management unless he or she be a Life Governor, or an annual subscriber of not less than two guineas a year. Thirdly, the Court summoned on the occasion when your reporter came was not the "annual," but a quarterly, in which the business on the agenda paper was merely the formal receiving of the "Quarterly Statement of Accounts." These quarterly statements are combined at the end of each financial year, and presented to the Annual Court in April, printed and distributed to all donors and subscribers, and are thus made public. I may also say that whenever there is any business to be transacted with which the public generally should be made acquainted, facilities will be always readily afforded for the purpose, especially in admitting a reporter from the office of THE HOSPITAL.

